# Predictors of early post ischemic stroke apathy and depression: a cross-sectional study

**DOI:** 10.1186/1471-244X-13-164

**Published:** 2013-06-05

**Authors:** Song-ran Yang, Ping Hua, Xin-yuan Shang, Rong Hu, Xiao-en Mo, Xiao-ping Pan

**Affiliations:** 1 Department of Neurology, Guangzhou First People’s Hospital, Guangzhou Medical University, No. 1 Panfu Road, Guangzhou 510180, China; 2 Sun Yat-sen Memorial Hospital, Sun Yat-sen University, Guangzhou 510120, China

**Keywords:** Stroke, Apathy, Depression, Executive dysfunction

## Abstract

**Background:**

Apathy and depression are important neuropsychiatric disorders that can occur after a stroke but the etiology and risk factors are not well understood. The purpose of this study was to identify risk factors for apathy and depression following a stroke.

**Methods:**

Patients with an acute stroke who met the inclusion criteria were recruited from our hospital, and general information was recorded from patient charts. The Apathy Evaluation Scale, Clinician Version (AES-C) was used to evaluate these patients within 2 weeks after the stroke. The Montreal Cognitive Assessment (MoCA), mini-mental state examination (MMSE), Hamilton Depression Scale (HAMD), Mattis Dementia Rating Scale Initiation/Perseveration subset (MDRS I/P), Frontal Assessment Battery (FAB) and Stroop Color-Word Association Test were employed to evaluate emotion, cognitive function and executive function. The patients were divided into two groups: the apathy group and the non-apathy group. We also divided the patients into two groups based on whether or not they had post-stroke depression. The clinical characteristics and scores on the MoCA, MMSE, HAMD and MDRS I/P were compared between the apathy and non-apathy groups as well as between patients with and without depression. Logistic regression analysis was performed to identify risk factors for apathy and depression following a stroke.

**Results:**

A total of 75 patients with acute stroke were recruited. Of these, 25 (33.3%) developed apathy and 12 (16%) developed depression. Multivariate logistic regression analysis indicated that a history of cerebrovascular disease (OR: 6.45, 95% CI: 1.48-28.05, P = 0.013), low HbA1c (OR: 0.31, 95% CI: 0.12-0.81, P = 0.017) and a low MDRS I/P score (OR: 0.84, 95% CI: 0.74, 0.96, P = 0.010) were risk factors for post-stroke apathy. Additionally, multivariate logistic regression indicated that a low MDRS I/P (OR: 0.85, 95% CI: 0.75, 0.97, P = 0.015) was associated with post-stroke depression.

**Conclusions:**

Three risk factors for post-stroke apathy were identified as a history of cerebrovascular disease, low HbA1c and lower MDRS I/P scores. A low MDRS I/P score was also identified as a risk factor for post-stroke depression. These results may be useful to clinicians in recognizing and treating apathy and depression in patients after a stroke.

## Background

Apathy and depression are important neuropsychiatric symptoms that can occur after a stroke and may be the core symptoms in some stroke patients [1,2]. It has been reported that in the acute phase of a stroke, 15.2-71% of patients demonstrate apathy [3]. Three to six months after a stroke the incidence of apathy has been reported to be 26.7% versus 5.4% in controls [4].

Cognitive dysfunction has been found to be related to apathy [5,6]; however, some patients with moderate to severe dementia do not show apathy so cognitive dysfunction per se does not produce apathy [7]. Some patients with post-stroke depression develop apathy [5]; however, apathy should probably be regarded as different from depression and it requires distinct prognostic and therapeutic strategies. For example, it has been found that in patients with a stroke, crying and sadness were associated with a subjective feeling of depression whereas apathy was not [8]. The situation may be more complex, however, as a relationship between depression and apathy could develop over time. A recent study found that 3 months after a stroke there was no significant overlap between apathy and depression, but one year later there was a significant overlap [9]. One group of authors who reviewed the literature on post-stroke depression and apathy proposed that “post-stroke depression” is really comprised of two syndromes, affective or depressive post-stroke depression and apathetic post-stroke depression, and that they have different neuroanatomical mechanisms [10].

It is important to identify stroke patients who are apathetic and to make the distinction between apathy and depression for several reasons: apathy increases the burden on caregivers, apathetic patients have been found to require longer hospitalizations and patients with apathy tend to participate less in rehabilitation [4]. Apathy and depression are also treated differently.

Knowledge about the risk factors for post-stroke apathy and depression would make it less likely that a clinician would fail to recognize and treat them. The aim of this study was to explore the risk factors for post-stroke apathy and depression in the subacute stage of a stroke.

## Methods

This study was approved by the Institutional Review Board of Guangzhou First Municipal People’s Hospital (No. 024, 2010). Informed consent was obtained from all patients before the study. The scale assessments were done by a qualified psychiatric specialist and the testing was confirmed by a psychiatrist and a neurologist.

### Patients

We reviewed the charts of 270 patients hospitalized for an acute stroke between December 2010 and December 2011. After detail evaluation with inclusion and exclusion criteria, 75 patients were used for this study. Inclusion criteria were: patients with a diagnosis of ischemic stroke (IS) according to valid ICD-10 codes [11], the stroke had occurred within the previous 7 days, computerized tomography (CT) was performed to exclude cerebral hemorrhage, patients were conscious and their Glasgow Coma Scale (GCS) scores were 15, patients were able to cooperate with the testing, and their admission NIH Stroke Scale (NIHSS) score was ≤8. All patients had acute lacunar infarctions.

Exclusion criteria were: patients with a diagnosis or history of atrial fibrillation, those with a history of schizophrenia, depression, anxiety or other mental illness, including dementia, Parkinson’s disease and other neurodegenerative diseases, the development of symptoms of severe aphasia so that patients were unable to complete the evaluation, severe heart, liver or kidney dysfunction, malignancies or thyroid diseases, contraindications to magnetic resonance imaging (MRI) such as pacemaker implantation and claustrophobia, drug abuse or drug dependence, and refusal to participate in the study. Smoking was defined as: Non-smoker, Ex-smoker who used to smoke but had quit, Current smoker was defined as those who continued to smoke 1–19 cigarettes per day during the study period [12]. Drinking was defined as: (1) Non-drinker who did not have a history of drinking in the past or was an occasional drinker (< 1 drink, where 1 unit equals to 8 gram of alcohol per week), (2) Light drinker of 1–15 units per week, (3) Moderate drinker of 16–42 units per week, (4) Heavy drinker of > 42 units per week, (5) Ex-drinker who used to drink but had quit [13].

### Collection of general information and grouping

Patients who met the inclusion criteria were recruited and their clinical information was recorded in detail. Data were recorded on hypertension, diabetes, a history of cerebral vascular disease and other chronic diseases. The NIHSS score had been recorded on admission. The evaluation of apathy was carried out using the Apathy Evaluation Scale, clinician version (AES-C) within 2 weeks after onset of the stroke. The AES was developed by Marin et al. and its reliability has been shown to be satisfactory [14]. The diagnostic criteria for apathy in this study were based on the Marin scale [14] and Robert et al. [15]. These criteria were (1) a lack of motivation relative to the patient’s previous level of functioning or the standards of his/her age and culture, (2) the presence of at least one symptom from the following three domains: (i) diminished goal-directed behavior (lack of effort, dependency on others to structure activity); (ii) diminished goal-directed cognition (lack of interest or concern about one’s personal problems), or (iii) diminished concomitant to goal-directed behavior (unchanging affect, lack of emotional responsiveness), (3) the symptoms cause clinically significant distress or impairment in social and occupational functioning, and (4) the absence of a diminished level of consciousness or administration of substances such as narcotics or medications [15].

### Detection of emotion, cognitive function and executive function

Within 2 weeks after the stroke, the Montreal Cognitive Assessment (MoCA), mini-mental state examination (MMSE), Hamilton Depression Scale (HAMD), Mattis Dementia Rating Scale initiation/perseverance subscale (MDRS I/P), Frontal Assessment Battery (FAB) and Stroop Color Word Test were employed to evaluate emotion, cognitive function and executive function. The MDRS can be used to evaluate attention, initiation/perseveration (I/P), construction, conceptualization and memory. The reliability of the MDRS Chinese version has been validated [16]. The MDRS I/P is one part of the MDRS and includes language production and coherent motion, and is used to assess the ability to initiate and sustain motion, as these are closely related to executive function of the frontal lobe. In the Stroop Color Word Test, two cards were used for testing. Card A was the Stroop color card and Card B the color word card. The time to complete the evaluation with each card was recorded and the difference between them was calculated (B-A_time_). The number of correct answers was recorded for each card and the difference was calculated (B-A_number_). On the HAMD, >35 points was considered to be an indication of serious depression and >20 points as mild or moderate depression. A MoCA score of <25 but ≥14 was defined as mild cognitive impairment (MCI). The scale assessments for the FAB were performed by a qualified psychiatric specialist and the testing was confirmed by a psychologist and a neurologist.

According to the Diagnostic and Statistical Manual of Mental Disorders (DSM IV), the criteria for major depression area depressed state of mind for most of the day, and at least four of the following: (1) loss of interest in or no pleasant feelings about activities, (2) the loss of energy or fatigue, (3) psychomotor retardation or agitation, (4) feelings of worthlessness, (5) diminished ability to think or concentrate, (6) recurrent thoughts of death, (7) sleep disorders such as insomnia or sleeping too much, and (8) significant weight loss or weight gain. The criteria for severity include impaired social functioning and pain or adverse consequences for the depressed individual. The symptom criteria and the severity criteria must have lasted for at least 2 weeks. The symptoms cannot be due to substances or a general medical condition.

According to the findings on T2-weighted images, the infarct lesions were located in the bilateral frontal lobes, temporal lobes, parietal lobe, occipital lobe, basal ganglia, corona radiata or brain stem.

The AES-C consists of 18 items which provide an index of overt goal-directed behavior, goal-related cognitions, and goal-related emotional responses. Factor analysis using principal component analysis was performed. The Kaiser-Meyer-Olkin (KMO) score for the AES-C was 0.84, indicating sampling adequacy (≥.50). Bartlett’s test of sphericity was statistically significant. The overall Cronbach’s alpha for internal consistency of the 18-item instrument was 0.87. HbA1c shows the average level of blood glucose over the previous three months and < 5.6% is considered normal.

### Statistical analysis

Comparison between the patients with and without post-stroke apathy and between those with and without post-stroke depression was conducted using the independent two-sample *t* test for continuous variables. The chi-square/Fisher’s exact test was used for categorical variables; the data are presented as numbers (percentages). Because multiple measurements were employed to evaluate emotion, cognitive function and executive function, Bonferroni corrections were used to reduce the chance of obtaining false-positive results. Univariate logistic regression analysis was performed to analyze the odds ratios of significant factors associated with patients with post-stroke apathy and post-stroke depression. Variables having a p value <0.2 in the univariate analysis were selected and evaluated by multivariate logistic regression models with the conditional forward selection method. All statistical assessments were two-tailed and p < 0.05 was considered statistically significant. Statistical analyses were performed using SPSS 15.0 statistical software (SPSS Inc, Chicago, IL).

## Results

Twenty-five (33.3%) of the 75 enrolled patients (28 women and 47 men) were diagnosed as having post-stroke apathy. The mean age of these patients was 66.7 ± 9.3 years (range, 48 to 84 years). Table 1 summarizes the demographic and clinical characteristics of the 25 patients with post-stroke apathy and the 50 patients without post-stroke apathy. There were significant differences between the two groups in age, education, frontal lesion location and HbA1c levels (p < 0.05). Twelve of the 75 patients (16%) had post-stroke depression. Table 2 summarizes the demographic and clinical characteristics of the patients with and without post-stroke depression. There was a significant difference between the two groups if the lesion was located in the temporal lobe (p = 0.048).

**Table 1:** Comparison of demographics, anthropometric characteristics, and hormonal and metabolic features between patients with and without post-stroke apathy

	**With apathy group**	**Without apathy group**	**p-value**
	**(n = 25)**	**(n = 50)**	
Age (yrs)^1^	70.7 ± 9.0	64.7 ± 8.9	0.007*
Gender, n (%)^2^			
Male	15 (60.0)	32 (64.0)	0.736
Female	10 (40.0)	18 (36.0)	
Education (yrs)	6.3 ± 5.3	9.0 ± 4.0	0.017*
Lesion location, n (%)^2^			
Frontal	12 (40.0)	10 (20.0)	0.012*
Temporal	6 (24.0)	4 (8.0)	0.075
Parietal	1 (4.0)	5 (10.0)	0.657
Occipital	1 (4.0)	4 (8.0)	0.659
Basal ganglia	18 (72.0)	35 (70.0)	0.858
Cerebellum	1 (4.0)	4 (8.0)	0.659
Brain stem	11 (44.0)	16 (32.0)	0.307
Corona radiata	6 (24.0)	4 (8.0)	0.075
Medical history, n (%)^2^			
Cerebrovascular disease	12 (48.0)	13 (26.0)	0.057
Hypertension	14 (56.0)	35 (70.0)	0.230
Diabetes mellitus	4 (16.0)	13 (26.0)	0.330
Smoking, n (%)^2^			0.703
Non-smoker	12 (48.0)	27 (56.0)	
Ex-smoker	3 (12.0)	3 (4.0)	
Current smoker	10 (40.0)	20 (40.0)	
Drinking, n (%)^2^			0.341
Non-drinker	17 (68.0)	38 (76.0)	
Light drinker	4 (16.0)	2 (4.0)	
Moderate drinker	3 (12.0)	8 (16.0)	
Heavy drinker	0 (0.0)	0 (0.0)	
Ex-drinker	1 (4.0)	2 (4.0)	
SBP (mmHg)^1^	148.7 ± 19.5	155.0 ± 21.1	0.215
DBP (mmHg)^1^	78.9 ± 12.8	86.8 ± 18.2	0.055
Heart rate (bpm)^1^	72.9 ± 12.7	74.4 ± 11.6	0.616
Total cholesterol (mmol/L)^1^	4.7 ± 0.9	5.0 ± 0.9	0.227
Triglycerides (mmol/L)^1^	1.4 ± 0.6	1.9 ± 1.3	0.081
LDL-C (mmol/L)^1^	2.7 ± 0.6	2.9 ± 0.7	0.340
HDL-C (mmol/L)^1^	1.1 ± 0.3	1.1 ± 0.3	0.578
HbA1c (%)^1^	5.8 ± 0.9	6.8 ± 1.7	0.018*
NIHSS^1^	2.4 ± 2.3	2.2 ± 2.3	0.612

*Indicates a significant difference between patients with and without post-stroke apathy, p < 0.05.

p values are from ^1^independent two sample tests and ^2^chi-square/Fisher’s exact tests.

Data are displayed as mean ± standard deviation and number (percentage).

Abbreviations: *SBP* Systolic blood pressure, *DBP* Diastolic blood pressure, *LDL-C* Low-density lipoprotein cholesterol, *HDL-C* High-density lipoprotein cholesterol, *HbA1c* Hemoglobin A1c, *NIHSS* National institutes of health stroke scale.

**Table 2:** Comparison of demographics, anthropometric characteristics, and hormonal and metabolic features between patients with and without post-stroke depression

	**With depression group**	**Without-depression group**	**p-value**
	**(n = 12)**	**(n = 63)**	
Age (yrs)^1^	66.0 ± 11.5	66.8 ± 8.9	0.780
Gender, n (%)^2^			0.346
Male	6 (50.0)	41 (65.1)	
Female	6 (50.0)	22 (34.9)	
Education (yrs)	9.6 ± 4.6	7.8 ± 4.5	0.223
Lesion location, n (%)^2^			
Frontal	3 (25.0)	19 (30.2)	1.000
Temporal	4 (33.3)	6 (9.5)	0.048*
Parietal	1 (8.3)	5 (7.9)	1.000
Occipital	2 (16.7)	3 (4.8)	0.179
Basal ganglia	10 (83.3)	43 (68.3)	0.491
Cerebellum	2 (16.7)	3 (4.8)	0.179
Brain stem	3 (25.0)	24 (38.1)	0.519
Corona radiata	9 (75.0)	43 (68.3)	0.745
Medical history, n (%)^2^			
Cerebrovascular disease	6 (50.0)	19 (30.2)	0.198
Hypertension	1 (8.3)	16 (25.4)	0.277
Diabetes mellitus	8 (66.7)	41 (65.1)	1.000
Smoking, n (%)^2^			0.893
Non-smoker	7 (58.3)	32 (50.8)	
Ex-smoker	1 (8.3)	5 (7.9)	
Current smoker	4 (33.4)	26 (41.3)	
Drinking, n (%)^2^	1 (8.3)	19 (30.2)	0.177
Non-drinker	10 (91.7)	44 (69.8)	
Light drinker	0 (0.0)	6 (9.5)	
Moderate drinker	0 (0.0)	11 (17.5)	
Heavy drinker	0 (0.0)	0 (0.0)	
Ex-drinker	1 (8.3)	2 (3.2)	
SBP (mmHg)^1^	148.8 ± 24.0	153.7 ± 20.1	0.454
DBP (mmHg)^1^	84.2 ± 16.2	84.1 ± 17.1	0.982
Heart rate (bpm)^1^	75.3 ± 12.7	73.7 ± 11.8	0.659
Total cholesterol (mmol/L)^1^	5.1 ± 1.0	4.8 ± 0.9	0.391
Triglycerides (mmol/L)^1^	1.6 ± 0.7	1.8 ± 1.2	0.662
LDL-C (mmol/L)^1^	3.0 ± 0.7	2.8 ± 0.7	0.383
HDL-C (mmol/L)^1^	1.2 ± 0.3	1.1 ± 0.3	0.488
HbA1c (%)^1^	6.1 ± 1.4	6.5 ± 1.6	0.449
NIHSS^1^	2.6 ± 2.7	2.2 ± 2.2	0.548

*Indicates a significant difference between patients with and without post-stroke depression, p < 0.05.

p values are from ^1^independent two sample tests and ^2^ chi-square/Fisher’s exact tests.

Data are displayed as mean ± standard deviation and number (percentage).

Abbreviations: *SBP* Systolic blood pressure, *DBP* Diastolic blood pressure, *LDL-C* Low-density lipoprotein cholesterol, *HDL-C* High-density lipoprotein cholesterol, *HbA1c* Hemoglobin A1c, *NIHSS* National Institutes of health stroke scale.

Table 3 shows the assessments of cognition and depression for the patients with and without post-stroke apathy. There were significant differences in MMSE, MoCA, and MDRS I/P scores between the two groups (p < 0.01). Patients without post-stroke apathy had significantly higher scores on the MMSE, MoCA, and MDRS I/P than did patients with post-stroke apathy. Assessments of cognition for patients with and without post-stroke depression are shown in Table 4. There was a significant difference in MDRS I/P scores between the two groups (p = 0.009). Patients without post-stroke depression had a significantly higher score on the MDRS I/P than did patients with post-stroke depression.

**Table 3:** Comparison of assessments of cognition and depression between patients with and without post-stroke apathy

	**With apathy group**	**Without apathy group**	**p-value**
	**(n = 25)**	**(n = 50)**	
MMSE score	23.0 ± 4.9	26.7 ± 2.8	<0.001*
MoCA score	17.3 ± 5.4	21.6 ± 4.9	0.001*
MDRS I/P	27.2 ± 5.1	31.7 ± 5.0	0.001*
FAB	12.3 ± 3.8	13.9 ± 2.9	0.050
HAMD	6.2 ± 5.3	4.2 ± 3.2	0.046*
Stroop color-naming			
B-A_time_	73.3 ± 47.9	59.3 ± 43.3	0.208
B-A_number_	−6.6 ± 8.2	−3.3 ± 6.9	0.070

* Indicates a significant difference between patients with and without post-stroke apathy, p < 0.01.

p values are from independent two sample tests and data are displayed as mean ± standard deviation.

Bonferroni corrections were used to reduce the chance of obtaining false-positive results.

Abbreviations: *MMSE* Mini-mental state examination, *MoCA* Montreal cognitive assessment, *MDRS I/P* Mattis dementia rating scale initiation/preservation subset, *FAB* Frontal assessment battery, *HAMD* Hamilton depression scale.

**Table 4:** Comparison of assessments of cognition between patients with and without post-stroke depression

	**With depression group**	**Without-depression group**	**p-value**
	**(n = 12)**	**(n = 63)**	
MMSE score	24.6 ± 4.3	25.6 ± 4.0	0.426
MoCA score	18.0 ± 5.2	20.6 ± 5.4	0.125
MDRS I/P	26.5 ± 5.7	30.9 ± 5.1	0.009*
FAB	13.3 ± 3.3	13.4 ± 3.3	0.976
Stroop color-naming			
B-A_time_	73.8 ± 59.6	62.1 ± 42.0	0.412
B-A_number_	−5.8 ± 9.9	−4.1 ± 6.9	0.458

* Indicates a significant difference between patients with and without post-stroke apathy, p < 0.01.

p values are from independent two sample tests and data are displayed as mean ± standard deviation.

Bonferroni corrections were used to reduce the chance of obtaining false-positive results.

Abbreviations: *MMSE* Mini-mental state examination, *MoCA* Montreal cognitive assessment, *MDRS I/P* Mattis dementia rating scale initiation/preservation subset, *FAB* Frontal assessment battery.

Table 5 presents the results of univariate and multivariate analysis to determine the factors potentially associated with post-stroke apathy. The univariate logistic regression model indicated that the following factors were significantly associated with post-stroke apathy: age, education, frontal lesion location, HbA1c, MMSE, MoCA and MDRS I/P (P < 0.05). Multivariate logistic regression indicated that a history of cerebrovascular disease (OR: 6.45, 95% CI: 1.48-28.05, P = 0.013), low HbA1c (OR: 0.31, 95% CI: 0.12-0.81, P = 0.017) and low MDRS I/P scores (OR: 0.84, 95% CI: 0.74, 0.96, P = 0.010) were associated with post-stroke apathy. In addition, the results of multivariate logistic regression indicated that a low MDRS I/P (OR: 0.85, 95% CI: 0.75, 0.97, P = 0.015) was also associated with post-stroke depression (Table 6). Table 7 presents the result of univariate multinomial logistic regression using patients without apathy and depression (n = 44) as the reference category. Table 7 had shown an increased risk of patients without apathy and with depression for temporal (OR = 10.50, 95% CI: 1.15–95.91), cerebellum (OR = 10.50, 95% CI: 1.15–95.91) and higher HAMD (OR = 8.01, 95% CI: 1.27–50.73). Risk of patients with apathy and without depression was also higher for older (OR = 1.12, 95% CI: 1.04–121), less education (OR = 0.73, 95% CI: 0.61–0.88), frontal (OR = 6.19, 95%CI: 1.88–20.34), brain stem (OR = 3.28, 95% CI: 1.07–10.02), lower MMSE score (OR = 0.72, 95%CI: 0.59–0.87), lower MoCA score (OR = 0.82, 95%CI: 0.73–0.0.93), lower MDRS I/P (OR = 0.85, 95%CI: 0.75–0.95), lower MDRS I/P (OR = 0.85, 95%CI: 0.75–0.95), and lower FAB (OR = 0.81, 95%CI: 0.69–0.96). The results of multivariate multinomial logistic regression were not estimable because of small sample size.

**Table 5:** Determinants of significant risk factors associated with post-stroke apathy (n = 75)

	**Univariate**		**Multivariate**	
	**OR (95% CI)**	**p-value**	**OR (95% CI)**	**p-value**
Age (yrs)	1.08 (1.02, 1.15)	0.011*		
Gender				
Female vs. Male	1.19 (0.44, 3.18)	0.736		
Education (yrs)	0.87 (0.77, 0.98)	0.021*		
Frontal				
Yes vs. no	3.69 (1.30, 10.52)	0.014*		
Temporal				
Yes vs. no	3.63 (0.92, 14.34)	0.066		
Parietal				
Yes vs. no	0.38 (0.04, 3.40)	0.383		
Occipital				
Yes vs. no	0.48 (0.05, 4.53)	0.521		
Basal ganglia				
Yes vs. no	1.10 (0.38, 3.18)	0.858		
Cerebellum				
Yes vs. no	0.48 (0.05, 4.53)	0.521		
Brain stem				
Yes vs. no	1.67 (0.62, 4.49)	0.309		
Corona radiata				
Yes vs. no	1.63 (0.55, 4.84)	0.378		
Cerebrovascular disease				
Yes vs. no	2.63 (0.96, 7.20)	0.060	6.45 (1.48, 28.05)	0.013*
Hypertension				
Yes vs. no	0.55 (0.20, 1.48)	0.232		
Diabetes mellitus				
Yes vs. no	0.54 (0.16, 1.88)	0.334		
Smoking				
Yes vs. no	1.27 (0.49, 3.33)	0.624		
Drinking				
Yes vs. no	1.49 (0.52, 4.31)	0.462		
SBP (mmHg)	0.99 (0.96, 1.01)	0.214		
DBP (mmHg)	0.96 (0.93, 1.00)	0.059		
Heart rate (bpm)	0.99 (0.95, 1.03)	0.610		
Total cholesterol (mmol/L)	0.71 (0.40, 1.24)	0.226		
Triglycerides (mmol/L)	0.49 (0.22, 1.07)	0.074		
LDL-C (mmol/L)	0.69 (0.32, 1.48)	0.335		
HDL-C (mmol/L)	0.61 (0.11, 3.39)	0.572		
HbA1c (%)	0.44 (0.20, 0.97)	0.043*	0.31 (0.12, 0.81)	0.017*
NIHSS	1.06 (0.86, 1.30)	0.606		
MMSE score	0.77 (0.66, 0.90)	0.001*		
MoCA score	0.85 (0.77, 0.95)	0.003*		
MDRS I/P	0.85 (0.76, 0.94)	0.001*	0.84 (0.74, 0.96)	0.010*
FAB	0.87 (0.75, 1.00)	0.057		
HAMD	1.13 (1.00, 1.27)	0.059		
Stroop color-naming				
B-A_time_	1.01 (1.00, 1.02)	0.211		
B-A_number_	0.94 (0.88, 1.01)	0.087		

*Significance factor, p < 0.05.

Abbreviations: *SBP* Systolic blood pressure, *DBP* Diastolic blood pressure, *LDL-C* Low-density lipoprotein cholesterol, *HDL-C* High-density lipoprotein cholesterol, *HbA1c* Hemoglobin A1c, *NIHSS* National Institutes of health stroke scale, *MMSE* Mini-mental state examination, *MoCA* Montreal cognitive assessment, *MDRS I/P* Mattis dementia rating scale initiation/preservation subset, *FAB* Frontal assessment battery, *HAMD* Hamilton depression scale.

**Table 6:** Determinants of significant risk factors associated with post-stroke depression (n = 75)

	**Univariate**		**Multivariate**	
	**OR (95% CI)**	**p-value**	**OR (95% CI)**	**p-value**
Age (yrs)	0.99 (0.93, 1.06)	0.777		
Gender				
Female vs. Male	1.86 (0.54, 6.47)	0.327		
Education (yrs)	1.09 (0.95, 1.26)	0.223		
Frontal				
Yes vs. no	0.77 (0.19, 3.17)	0.720		
Temporal				
Yes vs. no	4.75 (1.10, 20.57)	0.037*		
Parietal				
Yes vs. no	1.06 (0.11, 9.92)	0.963		
Occipital				
Yes vs. no	4.00 (0.59, 27.02)	0.155		
Basal ganglia				
Yes vs. no	2.33 (0.67, 11.61)	0.304		
Cerebellum				
Yes vs. no	4.00 (0.59, 27.02)	0.155		
Brain stem				
Yes vs. no	0.54 (0.13, 2.20)	0.391		
Corona radiata				
Yes vs. no	1.39 (0.34, 5.72)	0.643		
Cerebrovascular disease				
Yes vs. no	2.36 (0.66, 8.11)	0.189		
Hypertension				
Yes vs. no	1.07 (0.29, 3.97)	0.916		
Diabetes mellitus				
Yes vs. no	0.27 (0.03, 2.23)	0.223		
Smoking				
Yes vs. no	0.47 (0.21, 2.57)	0.633		
Drinking				
Yes vs. no	0.21 (0.03, 1.75)	0.149		
SBP (mmHg)	0.99 (0.96, 1.02)	0.449		
DBP (mmHg)	1.00 (0.96, 1.04)	0.982		
Heart rate (bpm)	1.01 (0.96, 1.07)	0.654		
Total cholesterol (mmol/L)	1.36 (0.68, 2.71)	0.386		
Triglycerides (mmol/L)	0.86 (0.44, 1.68)	0.658		
LDL-C (mmol/L)	1.52 (0.60, 3.82)	0.378		
HDL-C (mmol/L)	2.06 (0.27, 15.48)	0.483		
HbA1c (%)	0.82 (0.49, 1.37)	0.450		
NIHSS	1.09 (0.83, 1.42)	0.543		
MMSE score	0.94 (0.82, 1.09)	0.425		
MoCA score	0.92 (0.82, 1.03)	0.130		
MDRS I/P	0.85 (0.75, 0.97)	0.015*	0.85 (0.75, 0.97)	0.015*
FAB	0.99 (0.82, 1.21)	0.975		
Stroop color-naming				
B-A_time_	1.01 (0.99, 1.02)	0.409		
B-A_number_	0.97 (0.90, 1.05)	0.457		

*Significant factor, P < 0.05.

Abbreviations: *SBP* Systolic blood pressure, *DBP* Diastolic blood pressure, *LDL-C* Low-density lipoprotein cholesterol, *HDL-C* High-density lipoprotein cholesterol, *HbA1c* Hemoglobin A1c, *NIHSS* National Institutes of health stroke scale, *MMSE* Mini-mental state examination, *MoCA* Montreal cognitive assessment, *MDRS I/P* Mattis dementia rating scale initiation/preservation subset, *FAB* Frontal assessment Battery, *HAMD* Hamilton depression scale.

**Table 7:** The results of univariate multinomial logistic regression for determining the risk factors of post-stroke apathy or depression alone

	**OR (95% CI)**		
	**Without apathy/with depression**	**With apathy/without depression**	**With apathy and depression**
	**(n = 6)**	**(n = 19)**	**(n = 6)**
Age (y)	1.04 (0.94, 1.15)	1.12 (1.04, 1.21)*	1.01 (0.91, 1.17)
Gender			
Male vs. Female	0.23 (0.04, 1.43)	0.64 (0.21, 1.95)	0.93 (0.15, 5.72)
Education (y)	0.87 (0.61, 1.11)	0.73 (0.61, 0.88)*	1.17 (0.94, 1.46)
Frontal			
Yes vs. no	2.25 (0.35, 14.49)	6.19 (1.88, 20.34)*	0.90 (0.09, 8.80)
Temporal			
Yes vs. no	10.50 (1.15, 95.91)*	5.60 (0.93, 33.77)	10.05 (1.15, 95.91)*
Parietal			
Yes vs. no	2.00 (0.19, 21.62)	0.56 (0.06, 5.33)	Not estimable
Occipital			
Yes vs. no	2.73 (0.24, 31.56)	Not estimable	2.73 (0.24, 31.56)
Basal ganglia			
Yes vs. no	2.33 (0.25, 21.89)	1.01 (0.32, 3.22)	2.33 (0.25, 21.89)
Cerebellum			
Yes vs. no	10.50 (1.15, 95.91)*	1.67 (0.10, 13.70)	Not estimable
Brain stem			
Yes vs. no	2.39 (0.42, 13.40)	3.28 (1.07, 10.02)*	Not estimable
Corona radiata			
Yes vs. no	0.48 (0.08, 2.61)	1.01 (0.32, 3.22)	Not estimable
Cerebrovascular disease			
Yes vs. no	3.40 (0.59, 19.54)	3.06 (0.98, 9.60)	3.40 (0.59, 19.54)
Hypertension			
Yes vs. no	2.33 (0.25, 21.89)	0.64 (0.21, 1.95)	0.47 (0.08, 2.61)
Diabetes mellitus			
Yes vs. no	Not estimable	0.45(0.11, 1.81)	0.48 (0.05, 4.49)
Smoking			
Yes vs. no	0.55 (0.09, 3.31)	1.22 (0.41, 3.57)	1.10 (0.20, 6.03)
Drinking			
Yes vs. no	Not estimable	1.56 (0.50, 4.88)	0.53 (0.06, 5.05)
SBP (mmHg)	0.99 (0.94, 1.03)	0.98 (0.96, 1.01)	0.98 (0.94, 1.03)
DBP (mmHg)	1.01 (0.97, 1.05)	0.97 (0.93, 1.01)	0.97 (0.90, 1.03)
Heart rate (bpm)	1.01 (0.94, 1.09)	0.99 (0.94, 1.03)	1.01 (0.93, 1.08)
Total cholesterol (mmol/L)	1.58 (0.59, 4.19)	0.68 (0.36, 1.30)	0.95 (0.37, 2.48)
Triglycerides (mmol/L)	0.95 (0.46, 1.95)	0.49 (0.20, 1.18)	0.47 (0.12, 1.90)
LDL-C (mmol/L)	2.72 (0.73, 10.09)	0.79 (0.33, 1.90)	0.73 (0.19, 2.80)
HDL-C (mmol/L)	0.63 (0.03, 12.44)	0.26 (0.03, 2.22)	3.13 (0.22, 45.71)
HbA1c (%)	0.90 (0.54, 1.59)	0.48 (0.21, 1.09)	0.33 (0.08, 1.41)
NIHSS	1.06 (0.71, 1.57)	1.04 (0.82, 1.32)	1.14 (0.80, 1.61)
MMSE score	0.81 (0.63, 1.05)	0.72 (0.59, 0.87)*	0.77 (0.61, 0.98)*
MoCA score	0.85 (0.72, 1.01)	0.82 (0.73, 0.93)*	0.84 (0.71, 0.99)*
MDRS I/P	0.85 (0.71, 1.01)	0.85 (0.75, 0.95)*	0.74 (0.60, 0.92)*
FAB	0.87 (0.67, 1.12)	0.81 (0.69, 0.96)*	0.99 (0.73, 1.36)
HAMD	8.01 (1.27, 50.73)*	1.07 (0.82, 1.38)	11.16 (1.68, 74.05)*
Stroop color-naming			
B-A_time_	1.01 (0.99, 1.03)	1.01 (0.99, 1.02)	1.01 (0.99, 1.03)
B-A_number_	0.95 (0.85, 1.07)	0.93 (0.86, 1.01)	0.94 (0.84, 1.04)

*Significant factor, P < 0.05.

Reference group: Without apathy and depression (n = 44).

Abbreviations: *SBP* Systolic blood pressure, *DBP* Diastolic blood pressure, *LDL-C* Low-density lipoprotein cholesterol, *HDL-C* High-density lipoprotein cholesterol, *HbA1c* Hemoglobin A1c, *NIHSS* National Institutes of health stroke scale, *MMSE* Mini-mental state examination, *MoCA* Montreal cognitive assessment, *MDRS I/P* Mattis dementia rating scale initiation/preservation subset, *FAB* Frontal assessment battery, *HAMD* Hamilton depression scale.

## Discussion

### Risk factors for post-stroke apathy and depression

In this study, we compared 25 stroke patients who had apathy with 50 stroke patients who did not. The two groups were similar except that the apathy group was significantly older, had significantly fewer years of education, had a significantly higher proportion of frontal lobe lesions, and had a significantly lower level of HbA1c. The patients were assessed within 2 weeks of their stroke. Multivariate logistic regression analysis revealed three risk factors for apathy: a history of cerebrovascular disease, a low HbA1c level and a low MDRS I/P score. We also compared 12 patients who had post-stroke depression with 63 who did not. The groups were similar except that a significantly higher proportion of patients with depression had temporal lobe lesions.

Our results differed from those of previous studies that had attempted to identify risk factors for post-stroke apathy. Sagen et al. [17] assessed patients within the first 2 weeks and at 4 months after the stroke, and, using multivariate logistic regression analysis, found that at 4 months after the stroke, somatic comorbidity was significantly associated with apathy and there was a borderline association with an HADS-D score ≥8 at admission. Caeiro et al. [3] assessed patients during the acute phase of a stroke (≤ 4 days) and stepwise regression analysis showed that cerebral hemorrhage, a low level of education and a right hemispheric lesion were predictors. Brodaty et al. [4] assessed patients at 3–6 months after a stroke and performed univariate analysis which showed that older age, more functional dependency and a lower score on the MMSE were associated with apathy. Mayo et al. [18] assessed apathy in 408 stroke patients by telephone interviews with caregivers at 1, 3, 6 and 12 months after the stroke and analyzed the data using the group-based trajectory method; they found that greater apathy was predicted by older age, poor cognitive status and low functional status. One possible reason for the conflicting findings is that assessments were carried out at different periods after the stroke. In our study, patients were assessed within 2 weeks of the stroke during the subacute phase. Different variables were assessed and different assessment instruments were used.

### MDRS I/P

We found that a decrease in the MDRS I/P score was a risk factor for both post-stroke apathy and post-stroke depression. Patients with one or both of these scoured lower than did the control group. Patients with post-stroke apathy without depression also had lower scores on the MMSE and the MoCA than did controls; however, they had lower HAMD scores than those without apathy but with depression, suggesting that these groups are really different.

In our study, abnormalities were not found in B-A_time_ and B-A_number_. The Stroop Color Word Test can be used to evaluate the degree of impairment of the lateral and inner-upper frontal lobe [19], but fails to specifically assess the impairment of the whole frontal lobe.

The frontal-subcortical circuit has been found to connect the frontal cortex and the striatum, globus pallidus, substantia nigra and thalamus, and functions as an important effector mechanism and interacts adaptively [20,21]. Dysfunction of the frontal-subcortical circuit is characterized by a reduction in executive function, apathy and impulsive behavior. Studies have revealed that most stroke patients with depression and executive dysfunction had infarct lesions in the frontal lobe [22,23]. A study of participants in the Age Gene/Environment Susceptibility-Reykjavik Study found that infarct lesions involving both cortex and subcortex were related to compromised processing and executive function [24]. Another study found that among patients with frontotemporal dementia, the severity of apathy was associated with atrophy in the right dorsolateral prefrontal cortex [25]. Brodaty et al. found that among stroke patients who underwent MRI there was a trend for apathy to be associated with the extent of MR signal abnormalities (hypertintensities) in the deep white matter and in the right hemisphere and right frontal-subcortical circuit [4].

### History of cerebrovascular disease

We also found that a history of cerebrovascular disease was a risk factor for post-stroke apathy. Angelelli et al. found that apathy was more frequently found at 6 months after a stroke than during the subacute phase [26]. Following cerebral infarction, the nerve fibers distant from the lesion may experience secondary damage. Animal studies have shown that connectivity remodeling was present in the cortex, hypothalamus, striatum, hippocampus and tissues surrounding the lesions following a stroke [27-30]. We therefore speculate that one possible explanation for a history of cerebrovascular disease as a risk factor for post-stroke apathy is that infarcts and secondary damage to nerve fibers following a stroke may impair the frontal-subcortical circuit and connectivity remodeling. Alexopoulos et al. [31] postulated two broad hypotheses for the mechanism of vascular depression. The first hypothesis was that small lesions disrupting critical neural pathways might precipitate vascular depression. The second was that an accumulation of lesions exceeding a threshold predisposes to depression. The second hypothesis, which is a “threshold hypothesis,” is most applicable to patients who have neurologically silent lesions or an old stroke. Hama et al. [32] investigated the correlation between damage to the basal ganglia or frontal lobe and depression (both affective and apathetic dimensions) in a study of 243 stroke patients examined with CT, and suggested that affective depression was associated with left frontal lobe damage whereas apathetic depression was associated with damage to the basal ganglia in both hemispheres. Our finding that there was a tendency for a larger proportion of patients in the apathy group to have a history of cerebrovascular disease compared with the non-apathy group suggests that there was a larger proportion of recurrent stroke cases in the apathy group compared with the non-apathy group, and that our finding supports the threshold hypothesis. Cognitive function was lower in the apathy group compared with the non-apathy group. These findings related to a history of cerebrovascular disease and cognitive function suggest that repeated ischemic strokes damage brain function bit by bit, leading to both apathy and cognitive dysfunction.

### HbA1c

We found that the level of HbA1c was also a risk factor for post-stroke apathy. Padala et al. [33] found that apathy was prevalent in patients with diabetes without depression. The mean HbA1c level was 0.66% lower for apathetic patients as compared to non-apathetic ones. Apathy may have a negative impact on self-care behaviors and diabetes control [33]. To the best of our knowledge, this is the first time that this variable has been associated with post-stroke apathy. Because of small sample size, however, it is very likely that the statistically significant relationship between HbA1c and post-stroke apathy that we found was due to chance. Therefore, this possible relationship needs to be explored further in larger studies before conclusions can be drawn.

Notably, results from our additional logistic regression analysis on patients separated into four categories, using patients without apathy and depression as the reference category, showed that most findings are largely similar; however, this additional analysis is limited by the lack of multivariate analysis due to small sample size. In this limited analysis, HbA1c is not a risk factor for post-stroke apathy, and new brain areas had also shown significant difference such as brain stem for post-stroke apathy and cerebellum for post-stroke depression. Thus, this highlights the importance of having more studies with larger sample size in order to confirm our findings.

### Limitations

Our study had several limitations. The sample size of 75 stroke patients was small and the study was carried out at only one medical center with the testing instruments used being selected based on the availability of department and hospital team support. Also, there was no further investigation of the influence of damage in the cerebral regions on apathy or on the influence of impaired connectivity in each cerebral region on apathy. In addition, we excluded patients with severe aphasia because they could not complete the evaluation, and this might limit the generalization of our findings. Moreover, as pointed out by Mayo et al. [18], based on modern psychometric standards, there is no available measurement of apathy that would be considered optimal. Lastly, we attempted to identify “risk factors” for apathy or depression alone; however, results of multivariate multinomial logistic regression were not estimable due to small sample size.

## Conclusions

A history of cerebrovascular disease and a low MDRS I/P score appeared to be predictors of post-stroke apathy in the subacute phase. A low MDRS I/P score was also identified as a predictor of post-stroke depression. Our results suggested that a low HbA1c level might also be a predictor of post-stroke apathy but, because of small sample size, we believe that this finding may be explained by chance. We speculate that many risk factors may lead to repeated ischemic stroke events, so that eventually the accumulation of lesions exceeds a threshold which then results in apathy as well as depression and cognitive disorders. These findings may help clinicians recognize and treat apathy and depression in patients after a stroke; however, before our findings become useful in a clinical setting, they would need to be confirmed in larger studies.

## Competing interests

The authors declare that they have no competing interests.

## Authors’ contributions

SY guarantor of integrity of the entire study, study concepts, study design, definition of intellectual content, literature research, clinical studies, data analysis, statistical analysis, manuscript preparation, manuscript editing and review. PH guarantor of integrity of the entire study, study concepts, study design, definition of intellectual content, literature research, clinical studies, data analysis, statistical analysis, manuscript preparation, manuscript editing and review. XS definition of intellectual content, literature research, clinical studies, data acquisition, statistical analysis, manuscript preparation, manuscript editing. RH definition of intellectual content, literature research, clinical studies, data acquisition. XM definition of intellectual content, literature research, clinical studies, data acquisition. XP guarantor of integrity of the entire study, study concepts, study design, definition of intellectual content, literature research, clinical studies, statistical analysis, manuscript editing. All authors read and approved the final manuscript.

## Pre-publication history

The pre-publication history for this paper can be accessed here:

http://www.biomedcentral.com/1471-244X/13/164/prepub
